# Oleoylethanolamide supplementation improves mood and reduces fatigue in veterans with GWI in a 15-week randomized, double-blind, placebo-controlled exploratory clinical trial

**DOI:** 10.1038/s41598-026-35168-3

**Published:** 2026-01-09

**Authors:** Laila Abdullah, Andrew P. Keegan, Michael Hoffmann, James Baraniuk, Wendy Mack, Kimberly Sullivan, Cheryl Luis, Cheryl Rindfleisch, Claire J.C. Huguenard, Adam Cseresznye, Gregory J. Aldrich, James E. Evans, Daniel Paris, Dakota Helgager, Fiona Crawford, Michael Mullan

**Affiliations:** 1 Roskamp Institute, Sarasota, FL USA; 2 James A. Haley VA Hospital, Tampa, FL USA; 3 Georgetown University, Washington, DC USA; 4 Department of Population and Public Health Sciences, Keck School of Medicine, University of Southern California, Los Angeles, USA; 5 School of Public Health, Boston University, Boston, MA USA

**Keywords:** Gulf War Illness, Oleoylethanolamide, Fatigue, Mood and energy, Neuroscience, Medical research, Neurological disorders

## Abstract

Gulf War Illness (GWI) affects 32% of Veterans from the 1990–1991 Gulf War. It is characterized by fatigue, mood disturbances, pain and cognitive decrements, with no FDA-approved treatment. This exploratory randomized, double-blind, placebo-controlled trial evaluated the safety and efficacy of oleoylethanolamide (OEA) supplementation in 52 Veterans with GWI (mean age 59 ± 5 SD, 94% males and 79% White). Participants received 200 mg of OEA twice daily or a matching placebo for 10 weeks, followed by a 5-week open-label OEA extension. Outcomes included the Multidimensional Fatigue Inventory (MFI-20), abbreviated Profile of Mood States (POMS), Short-Form McGill Pain Questionnaire, Veterans RAND 36-Item Health Survey (SF-36 V), Repeatable Battery for the Assessment of Neuropsychological Status (RBANS) and CNS Vital Signs Test, administered at baseline and 10- and 15-weeks. A main effect of OEA for reducing fatigue (MFI-20, *p* ≤ 0.05) and improving the total mood disturbances score (POMS, *p* ≤ 0.05) was observed. Participants on OEA reported increased energy, emotional well-being, and social functioning (SF-36 V, *p* ≤ 0.05). No changes were seen in cognitive performance or pain. Oleoylethanolamide was well-tolerated with no serious adverse events. In summary, OEA was safe and improved fatigue and mood in Veterans with GWI. Larger trials are needed to confirm these benefits.

Registered under ClinicalTrials.gov Identifier no. NCT05252949 (first posted date: 23/02/2022).

**Supplementary Information:**

The online version contains supplementary material available at 10.1038/s41598-026-35168-3.

## Introduction

Thirty-five years have now elapsed since the 1990–1991 Gulf War (GW) conflict, yet nearly one-third of the 700,000 Veterans from this conflict continue to suffer from Gulf War Illness (GWI)^1,2^, a condition that is characterized by cognitive impairment, mood disturbance, and fatigue, all of which contribute to a significantly diminished quality of life^2^. These features result from a complex pathophysiology involving multiple organ systems and biological factors, such as bioenergetic deficits, metabolic dysregulation and chronic immune dysfunction^3,4^. Despite the significant prevalence of this condition among GW Veterans, there has been limited progress in developing effective interventions. Currently, no approved therapies exist that can improve the quality of life for Veterans with GWI, underscoring a critical gap in their care. As such, interventions are needed to help improve the general health and well-being of Veterans with GWI.

Oleoylethanolamide (OEA) is a peroxisome proliferator-activated receptor-alpha (PPARα) agonist^5^. While it is primarily known for regulating energy balance, appetite, and lipid metabolism^6–9^, OEA also influences key signaling pathways associated with mood regulation, cognition, and energy enhancement^10^. For instance, OEA is synthesized by the enterocytes in the small intestine, where it modulates the gut-vagal-brainstem axis^11^ through activation of PPARα in these enterocytes and neighboring cells^11,12^, which ultimately results in altered lipid metabolism and nutrient sensing, facilitating vagal afferent signaling through the nucleus tractus solitarius (NTS)^10^. There, synaptic inputs induce changes in immediate-early genes (IEGs)^13^, resulting in downstream modulation of circuits related to energy expenditure and emotional regulation^14,15^. Activation of IEG dampens inflammation and attenuates sickness behaviors that contribute to fatigue and low mood^16^. Through this mechanism, OEA is thought to exert anti-inflammatory and neuroregulatory effects, thereby improving energy balance, fatigue and mood^5,17,18^. Additionally, activation of PPARα by OEA enhances mitochondrial function and energy homeostasis, which is crucial for mitigating fatigue^19–21^. The cognitive benefits of OEA are believed to result from enhanced memory consolidation via emotional arousal, driven by autonomic signals transmitted through the NTS to the forebrain, which in turn activates noradrenergic neurons in the basolateral amygdala^22^.

Oleoylethanolamide and anandamide (AEA) are both members of the N-acylethanolamine (NAE) family and share degradative enzymes such as fatty acid amide hydrolase (FAAH)^23^. Although both OEA and AEA are shown to activate PPARα and transient receptor potential vanilloid 1; however, unlike AEA, OEA does not activate cannabinoid receptors^24–26^. Instead, the influence of OEA on the cannabinoid receptors appears to be indirect and is attributed to an increase in AEA by competing for degradation by FAAH^27^. As such, this indirect involvement of OEA in modulating the endocannabinoid system may help stabilize mood and alleviate fatigue^28–30^. Our preclinical mouse model studies have indicated that OEA can reduce fatigue and neurobehavioral features in mice exposed to an anti-nerve agent and pesticides (GW toxicants) implicated in GWI pathogenesis^31^. We therefore hypothesize that OEA will normalize mood and help mitigate fatigue and improve cognition in Veterans with GWI. To test this, we performed a single-site, randomized, double-blind, placebo-controlled clinical study to investigate whether OEA supplementation could reduce fatigue and improve mood, cognition, and overall well-being in Veterans with GWI.

## Materials and methods

### Study design

Using a single-site randomized, double-blind, placebo-controlled study design, 200 mg of OEA or a matching placebo was administered orally twice daily over a blinded period of 10 weeks, followed by an open label 5-week extension with OEA, allowing all study participants to take the active supplement. This study was conducted in accordance with the ethical principles outlined in the Declaration of Helsinki, and in compliance with the Good Clinical Practice (GCP) guidelines described in the International Conference on Harmonization (ICH) Guidelines. The Institutional Review Board (IRB) approval was obtained from the WIRB-Copernicus Group (IRB #: 20191414). All subjects were provided with oral and written information describing the nature, purpose, and duration of the study. Each subject provided written informed consent before any study-specific procedures were performed, in accordance with ICH GCP guidelines. There were 5 in-person clinic visits, including the screening visit and 3 phone call visits. Subject eligibility was determined by an in-person screening visit after completion of the consent process.

### Participant selection

The trial was registered under ClinicalTrials.gov Identifier no. NCT05252949 (first posted date: 23/02/2022). The recruitment began in May 2021 and ended in February 2024. Participants were recruited primarily from the state of Florida and others from around the USA who were willing to travel to the study site and met the study visit criteria. Participants were recruited online through IRB-approved advertisements and site-specific databases of former participants who had permitted re-contact by the research team. WEHealth was also utilized, which connects participants to clinical trials by deploying targeted outreach campaigns and advertisements through online outlets such as social media. Potential participants were directed to IRB-approved study flyers if they met the specific trial requirements. The trial inclusion criteria included a willingness to provide written consent, deployment to the GW theatre between August 1990 and July 1991 and a diagnosis of chronic multisymptom illness (CMI) consistent with the Centers for Disease Control and Prevention (CDC)^32^ and GWI using the Kansas GWI case definition^33^. Additionally, both sexes and all ethnic groups up to age 70 were eligible. Since preclinical studies suggested a potential role of OEA in satiety and weight loss^34^, weight limitations were between 50 and 200 kg (110lbs – 440lbs). The exclusion criteria for the study included a clinical diagnosis of medical or psychiatric conditions that would account for their symptoms or interfere with their ability to report their symptoms. Examples of these medical exclusions included the following: untreated chronic hypertension (defined as systolic blood pressure > 180 mmHg; diastolic blood pressure > 110 mmHg), myocardial infarction within 6 months of screening, renal failure, hepatic failure, and/or are recipients of chemotherapy. Other psychiatric conditions were also considered exclusionary. While reviewing the inclusion/exclusion criteria, medical history and medication use information, if a participant reported a major psychiatric condition (i.e., bipolar disorder, schizophrenia and major depression), the clinical investigator reviewed the information and requested additional information to assess the stability of the reported psychiatric condition. The clinical investigator made the determination of eligibility based on this information. No formal clinical interview or testing was completed. Additional exclusions for female subjects included being pregnant or nursing, or if a female subject was of childbearing age but was unwilling/unable to use birth control. Potential participants with contraindications, allergies, or sensitivities to OEA, olive oil, or excipients were also excluded. Poor venous access, current use of OEA supplement products within 30 days of screening or enrollment and having participated in another clinical trial involving dietary or pharmaceutical intervention within 90 days of screening were also exclusion criteria.

### Investigational product information

Oleoylethanolamide has been tested in several human clinical studies^7,35,36^. The FDA acknowledged receipt of a New Dietary Ingredient (NDI) notification on June 1, 2015. It is currently available from various sources as a dietary supplement on the open market for weight management. The supplement formulation for this study was not the product referenced in the NDI above. However, it contains GMP OEA in a capsule formulation of 200 mg of OEA to be taken twice a day by mouth (PO), with food. We therefore selected this dose to be consistent with this and other similar OEA dietary supplement dosages available currently. The excipients of the capsule formulation are rice bran extract, microcrystalline cellulose, silicon dioxide, and hydroxypropyl methylcellulose. Visually matching placebo capsules contain the same inactive ingredients present in the manufactured OEA capsules, except for any OEA compound. Oleoylethanolamide and matching placebo capsules were stored at temperatures between 15 and 32 ℃ in a locked and secured area, per ICH GCP guidelines.

### Randomization and blinding

Following a successful screening, each participant eligible for randomization was assigned a subject ID number at enrollment and all subsequent analyses were performed using the subject ID. The study pharmacists assigned study subjects to either the placebo or OEA group (1:1) using a non-stratified online block randomization program with a block of 4. The study coordinator dispensed the study product provided by the pharmacist to the participants at enrollment and at each study visit. At each study visit, a new bottle of the study product was dispensed, and the old bottle was collected for manual pill counting to help with assessing compliance. Investigators and study staff who conducted the study procedures were blinded to the randomization assignments. Only the pharmacists had access to the randomization code. The blind was broken only after the database lock was released. There were no serious adverse events that required blinding to be broken during the trial.

### Adverse events

All adverse events (AE) reported by the participants were recorded, regardless of whether they were considered related to the investigational product or not. Adverse events were classified into standardized terminology from the verbatim description according to the Medical Dictionary for Regulatory Activities (MedDRA version 12.1 or higher) coding dictionary, and reviewed by the designated safety committee. Grade and severity levels were recorded per Common Terminology Criteria for Adverse Events (CTCAE) guidelines. Adverse events were reviewed and assessed by the medical doctor on the study team for their severity, relationship to the Investigational Product, and whether the AE was expected or not. The medical doctor then determined the outcome and action taken for the subject.

### Study assessments

**Fig. 1:**
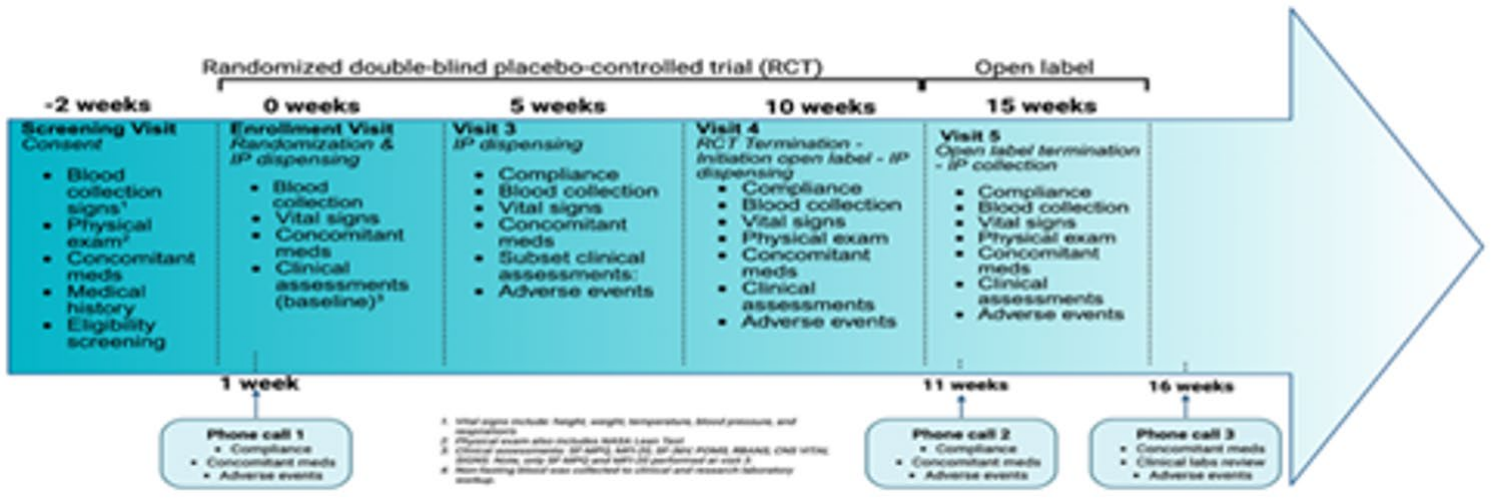
Study schedule of visits and data collection. This diagram shows the study timeline and procedures at each visit. These comprised of the MFI-20 test, CNS-vital signs, RBANS, abbreviated POMS and SF-36 V.

**Fig. 2:**
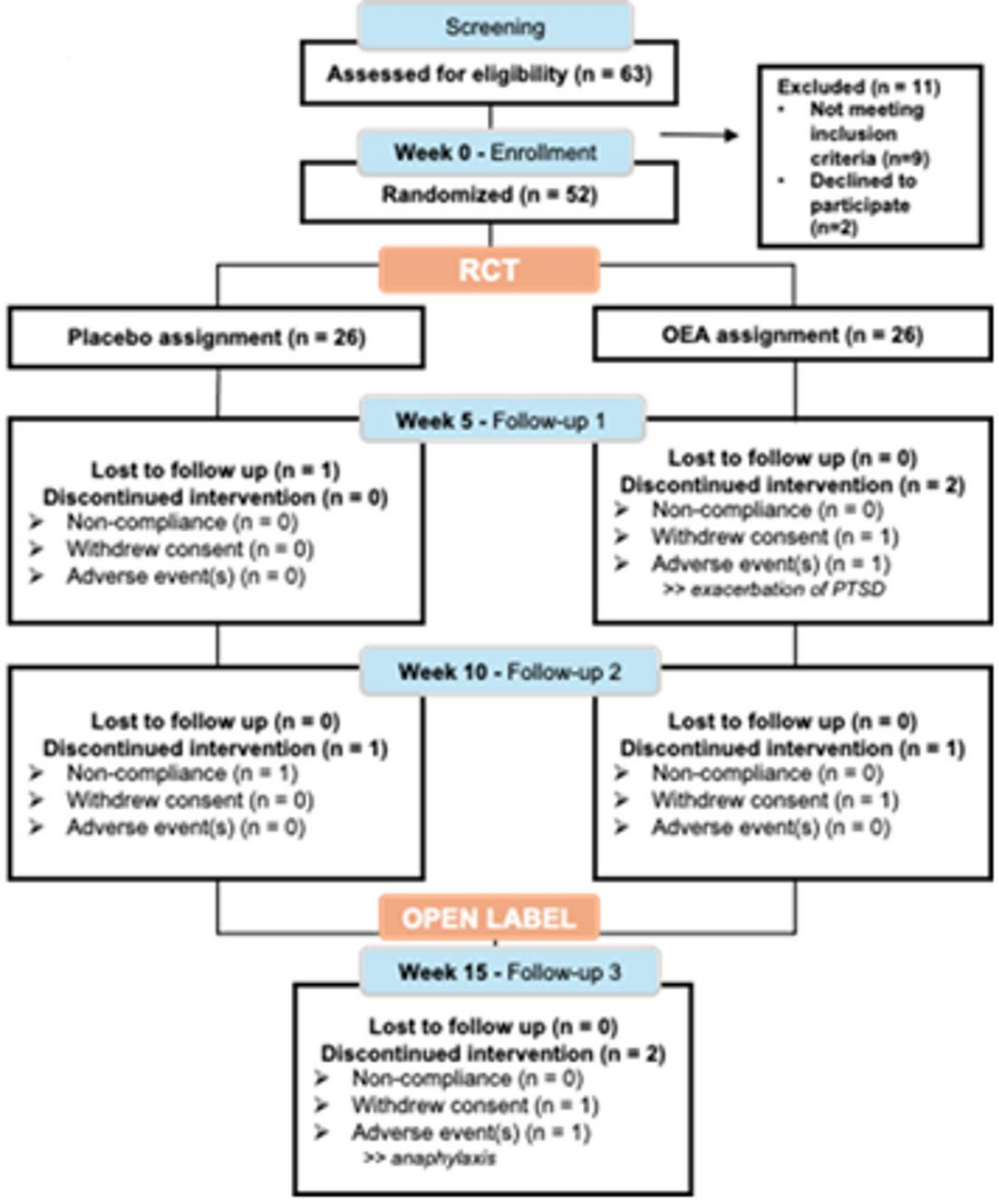
Consort flow diagram of the progress through the RCT and the open-label phases of a randomized, double-blind, and placebo-controlled clinical trial of the OEA supplementation. This diagram shows the flow of participants in each arm of the study. Twenty-six participants were randomized to each of the placebo and the OEA groups. One participant in the placebo group was lost to follow-up during the RCT. One placebo participant was non-compliant by the end of the RCT, and 1 withdrew consent in the OEA group.

Assessments included a complete physical exam and measurements of vital signs, including temperature, blood pressure, pulse and respiratory rate. The NASA Lean Test for orthostatic intolerance was also completed as previously described^37^. Clinical labs included standard renal and liver function tests (comprehensive metabolic panel and a complete blood count with differential) and were obtained from a blood draw, where a fasting state was not required to assess for any clinically significant abnormalities. These procedures were performed at baseline and at each in-person visit. Additionally, at each visit (in-person or by phone), questions were asked regarding how each participant felt using open-ended questions to collect potential adverse event information. The order of the study procedures for baseline and each visit generally consisted of consenting, review of the vitals signs, the NASA Lean Test, research and clinical blood draws, self-report assessments and then cognitive assessments as applicable. Concomitant medication logs captured any prescribed or other medication (e.g., over the counter, dietary supplement, and self-report of cannabis use) taken within 30 days prior to screening. The surveys included trial outcome measures consisting of standardized self-report instruments for fatigue, pain, and quality of life and objective cognitive testing was also performed (see Fig. 1 for the study flow-chart for all participants through the study-specific procedures conducted at each in-person visit) and Fig. 2 which presents the Consolidated Standards of Reporting Trials [CONSORT]).

The test for Fatigue was measured using the Multidimensional Fatigue Inventory (MFI-20) that measures five dimensions of fatigue: general, physical and mental fatigue as well as reduced activity and motivation^38^. Respondents use a scale ranging from 1 to 5 to self-report how aptly certain statements regarding fatigue represent their experiences. All scores range from 1 to 5 (1 = yes “that is true” where 5 is equivalent to no, “that is not true”). Positively phrased items were reverse scored so that a higher score represented more severe fatigue. Subcategories of fatigue on the MFI-20 questionnaire were scaled to 100^39^.

Mood-related outcomes were self-reported by participants using the abbreviated Profile of Mood States (POMS), which utilizes forty single-word affective state descriptors endorsed for the degree of severity and summed for six mood scales: tension, depression, fatigue, confusion, anger, and vigor^40^. Total mood disturbance (TMD) was scaled to 100 and was formed by subtracting the (positive) value of the vigor scale from the sum of the remaining five scales.

The general well-being of participants was assessed using a widely used health-related quality-of-life scale for Veterans developed by the RAND Corporation (Short-Form 36 Veterans version [SF36V])^41^. For this questionnaire, the respondents were asked to answer questions referring to the past 4 weeks. Survey questions within subscales were totaled to provide a summed score for each subscale or dimension, which included physical functioning, role limitations due to physical health, role limitations due to emotional problems, energy/fatigue, emotional well-being, social functioning, pain, and general health^42^. Briefly, questions from the survey within each of these categories were first rescaled to 100, then reverse coded so that the higher numbers indicated improvements, and finally, an average was calculated based on the number of questions in each category.

Although the complete McGill Questionnaire was used to assess self-report of pain for the first 8 participants, many participants found it too lengthy and did not complete the form, leaving many questions unanswered. Therefore, the protocol was amended to use the short-form McGill Questionnaire (SF-MPQ)^43^, which was used for the remainder of the study participants. This assessment utilizes fifteen descriptors (11 sensory, 4 affective) to describe current pain, as well as present the pain intensity index to assess total pain experience. Since participants completing the long MPQ are able to see all 78 descriptors, their choice of intensity may be slightly influenced by the larger context (vs. seeing only the short list). Therefore, we considered data from the first 8 participants to be missing and excluded them from the analyses.

Cognitive assessments were performed by trained study staff members after the collection of self-report surveys and included the CNS Vital Signs test, which is a battery of computer-based assessments of various cognitive functions, ranging from executive functioning to information processing speed^44^. The Repeatable Battery for the Assessment of Neuropsychological Status (RBANS) versions A and B were administered by qualified trained study staff. This test battery allows for the brief assessment of memory function, visuospatial skills, language fluency, naming and attention^45^.

### Plasma cytokine, low-density lipoprotein (LDL) and triglyceride analyses

All aspects of blood collection, processing and biomarker analysis were performed in a blinded manner. Non-fasting blood draws were conducted by trained phlebotomists and samples were collected in EDTA-containing vacutainers and centrifuged with 15 min of collection at 10 min at 3,200 x g at 4 °C, with a fast acceleration and a fast deceleration. Plasma cytokines analysis was performed using the Mesoscale Discovery (MSD) V-PLEX Viral Panel 2 (human) Kit, SECTOR (catalog #: K15346D-1), which included interferon gamma (IFN-), interleukin (IL)−4, IL-6, IL-10, IL-8 and Tumor necrosis factor alpha (TNF)-α as per manufacturer’s instructions. Plasma low-density lipoprotein (LDL) levels were quantified using the enzyme-linked immunosorbent assay (ELISA) from ThermoFisher Scientific (catalog #: L3486) and triglycerides (TG) levels were quantified using ELISA available from Cayman Chemicals (catalog #: 10010303) as per their respective manufacturers’ instructions.

### Plasma ethanolamide analyses

Sample preparation was based on the method by Palandra and colleagues^46^ with minor modifications. Briefly, 25 µL of plasma was spiked with 5 µL of 100 µg/mL deuterated ethanolamides (Cayman Chemical, USA) in methanol. Samples were allowed to equilibrate at room temperature for 10 min prior to protein precipitation with 125 µL of acetonitrile (ACN). After vortexing for 30 s, the samples were incubated at room temperature for 30 min to facilitate protein precipitation, followed by centrifugation at 10,889 × g for 15 min at room temperature. The resulting supernatant was transferred to autosampler vials with low-volume inserts and stored at −20 °C until liquid chromatography/mass spectrometry (LC/MS) analysis.

Chromatographic separation was performed using a Thermo Vanquish UPLC system (Thermo Fisher Scientific, USA) equipped with an Acquity UPLC BEH C18 column (1.7 μm, 1.0 × 50 mm; Waters, USA). The mobile phases consisted of Solvent A (10% ACN in water with 5 mM ammonium acetate and 0.1% acetic acid) and Solvent B (methanol with 5 mM ammonium acetate and 0.1% acetic acid). Isocratic elution was carried out at 80% B with a flow rate of 50 µL/min. Mass spectrometric detection was performed on a Thermo Orbitrap Exploris 240 mass spectrometer (Thermo Fisher Scientific, USA) operated in positive ion mode using targeted parallel reaction monitoring (PRM). The resolution was set to 45,000 (FWHM at m/z 200), with a fixed normalized collision energy of 34%, a standard AGC target, sheath gas at 25 arbitrary units (Arb), auxiliary gas at 5 Arb, ion transfer tube temperature at 320 °C, and vaporizer temperature at 75 °C. To monitor analytical performance and ensure data quality, a procedural standard was prepared by adding 5 µL of the 100 µg/mL OEA to 25 µL of water and 125 µL of ACN, mimicking the extraction conditions. This standard was injected alongside the study samples to evaluate peak quality and consistency.

Each analytical batch included external quality control (QC) samples to monitor batch-to-batch reproducibility. Coefficients of variation (CVs) were calculated for OEA across the nine batches to assess analytical precision. Additionally, blanks were processed and injected with each batch to check for carryover and background interference. Chromatograms corresponding to OEA and other ethanolamides were inspected in these blanks to confirm the absence of signal contamination. Data calculations were performed using the Quan Browser function within the Xcalibur software (ThermoFisher Scientific). All ethanolamides were calculated against the d2-OEA standard.

### Safety analysis

The incidence of adverse events (AEs) was summarized by severity and relationship to the study product as reported in the results section.

### Statistical analyses

The target sample size of 52 was determined based on precedent from similar pilot studies involving OEA. It was considered appropriate for an initial assessment of feasibility, safety, and preliminary effect trends^47^. All outcome measures for fatigue, pain, mood and quality of life and cognitive performance were considered exploratory. Given the exploratory nature of the study, a priori power calculations for the above outcome measures were not performed (see supplementary methods for *post-hoc* power calculations). Plasma cytokines, LDL, TG and ethanolamide concentrations were also evaluated as the key biological outcome measures. An intent-to-treat (ITT) design was used, and all participants completing all the study visits were included in the analysis. Descriptive statistics were used to characterize the sample. Between-group differences in demographics were assessed using either the Chi-square test or Student’s t-test, as appropriate. Outcome data were evaluated for normality and log-transformed where necessary to meet model assumptions.

Pre-planned analyses were performed with mixed linear models (MLM) to examine the main effect of OEA supplementation on the outcome measures (separately for each clinical assessment, cytokine, LDL, TG and ethanolamide), effects of time itself (incorporated as an indicator variable) and any interactions with time (fixed factors); participant-level random effects were included. These analyses included both the 5-week and 10-week randomized clinical trial (RCT) assessments, as well as, the subsequent 5-week open-label phase, to accommodate handling of unbalanced data, missing values, and complex variance-covariance structures. If the main effects of OEA intervention, time or the interaction between time and OEA intervention were found to be statistically significant, *post-hoc* analyses were performed using the paired t-test as complementary and exploratory comparisons to contextualize the MLM results and to examine within-group changes from baseline to each follow-up visit. The pre-planned paired t-test analysis was designed to assess within group changes in the 10-week RCT phase and also accommodate the 5-week open-label extension where the placebo group also received OEA. To account for multiple comparisons across key measures, including total scores from the MFI20, POMS, SF-MPQ, RBANS, and CNS Vital Signs tests, the Benjamini and Hochberg method was applied to control the false discovery rate (FDR). A two-sided alpha of 0.05 or less was used as a threshold for statistical significance in these corrected analyses. No additional corrections were applied to subscale scores or *post-hoc* paired t-tests, as these were considered hypothesis-generating for evaluating changes over time and intended to inform the design of future trials. Total numbers and specific grouped AEs were analyzed using the Chi-square test with continuity correction, as applicable. All analyses were performed using the SPSS version 29.0.

## Results

### Intervention with OEA reduces fatigue and improves mood over 15 weeks

There were no significant differences in baseline demographics or any of the clinical outcome measures, including MFI-20, POMS, CNS-Vital Signs test, RBANS and SF-MPQ questionnaires (*p* > 0.05, Table 1). There were no differences in weight between the OEA and the placebo groups at baseline or at any of the subsequent visits (*p* > 0.1). The average compliance with taking the study intervention was 91%.

**Table 1:** General demographics and baseline measures for the OEA and placebo groups.

General demographics	Placebo	OEA	*p*-value
	(*n* = 26)	(*n* = 26)	
Age (years) mean ± SD	59 ± 5	58 ± 5	0.46
Sex, n (%)			
Male	23 (89)	26 (100)	0.23
Female	3 (11)	0 (0)	
Race, n (%)			
White/Caucasian	22 (85)	19 (73)	0.50
Black/African American	04 (15)	06 (23)	
Other/Multiracial	00 (00)	01 (04)	
Ethnicity, n (%)			
Non-Hispanic/Latino	22 (85)	22 (85)	1.00
Hispanic/Latino	04 (15)	04 (15)	
Education, n (%)			
12 years (High School Diploma/GED)	03 (12)	03 (12)	0.40
13–15 years (Associate degree/ 2-year college/Other)	13 (50)	16 (62)	
16-years (Bachelor’s degree)	06 (23)	03 (12)	
17 + years (Advanced or professional degree)	04 (15)	04 (15)	
Weight (kg) mean ± SD	100 ± 21.4	98 ± 18.3	0.82
BMI ± SD	32.5 ± 5.1	30.2 ± 5.7	0.14
Cannabis use, n (%)	3 (12)	3 (12)	1.00
CNS vital sign test NCI ± SD	213 ± 30	211 ± 20	0.76
RBANS (sum of index) mean ± SD	439 ± 59.4	430 ± 47.2	0.56
MFI-20, mean ± SD	76 ± 17.3	70 ± 16.6	0.22
POMS, mean ± SD	27 ± 32.3	21 ± 32.6	0.52
*SF-McGill pain questionnaire, mean ± SD	14.4 ± 12.1	14.5 ± 11.6	0.98

*Data unavailable for 8 participants for whom the full version of this test was used. Note: No statistically significant differences were observed at baseline between the placebo and control groups (*p* > 0.05). Multifunctional Fatigue Inventory 20 (MFI20), abbreviated Profiles of Mood States (POMS) and Repeatable Battery for the Assessment of Neuropsychological Status RBANS), Neurocognitive index (NCI) and Short-form McGill Pain Questionnaire.

**Fig. 3:**
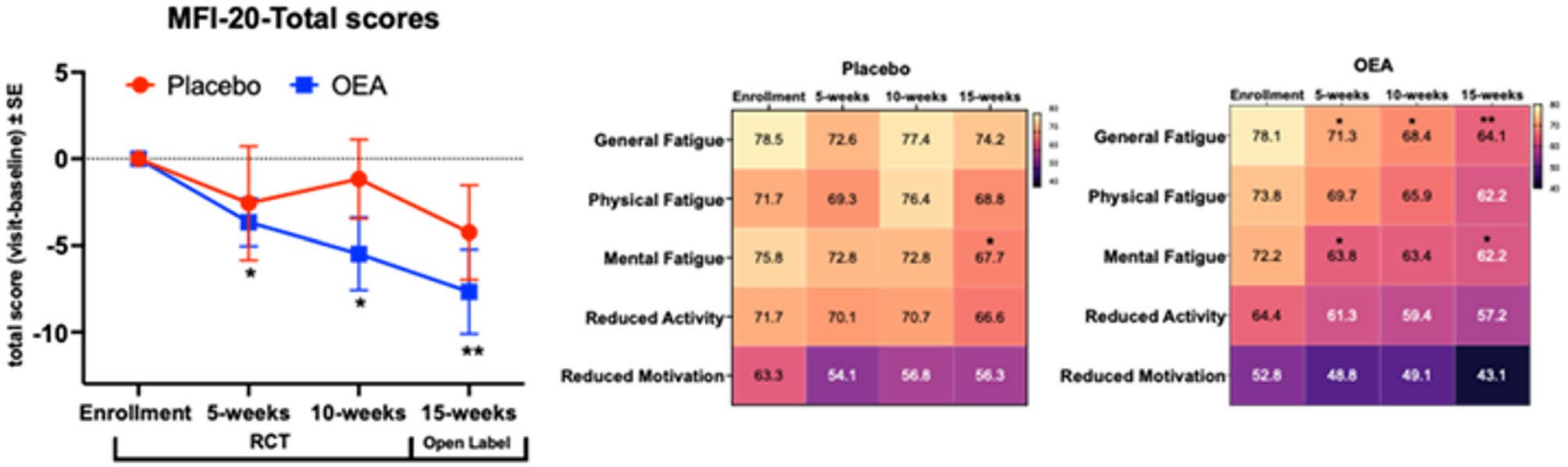
The MFI-20 total scores and sub-scale scores showed a reduction of fatigue among Veterans with GWI who received OEA supplement. (**A**) Following MLM regression analysis for the main effect (F_(1,180)_ = 6.5, *p* ≤ 0.05) of differences between the OEA and the placebo groups for the total MFI-20 score, paired t-tests (mean ± SE, placebo *n* = 23; OEA *n* = 20) were performed. Differences for each visit were significant in the OEA group compared to their baseline (t-value _(df =22)_ = 2.5, *p* = 0.02 for 5-weeks, t-value _(df =22)_ = 2.6, *p* = 0.02 for 10-weeks and t-value _(df =22)_ = 3.1, *p* = 0.005 for 15-weeks). (**B**) Heatmap of scores for each category. For general fatigue, physical fatigue and mental fatigue, scores were lower compared to the baseline scores in the OEA group over the RCT and the open label phases (general fatigue: t-value _(df =23)_ = 2.6, *p* = 0.01 for 5-weeks, t-value _(df =22)_ = 2.7, *p* = 0.01 for 10-weeks and t-value _(df =20)_ = 3.5, *p* = 0.002 for 15-weeks; mental fatigue: t-value _(df =23)_ = 2.5, *p* = 0.02 for 5-weeks, t-value _(df =22)_ = 1.8, *p* = 0.082 for 10-weeks and t-value _(df =20)_ = 2.5, *p* = 0.02 for 15-weeks; reduced activity: t-value _(df =23)_ = 1.4, *p* = 0.18 for 5-weeks, t-value _(df =22)_ = 1.4, *p* = 0.17 for 10-weeks and t-value _(df =22)_ = 1.7, *p* = 0.10 for 15-weeks and reduced motivation: t-value _(df =22)_ = 0.68, *p* = 0.50 for 5-weeks, t-value _(df =22)_ = 1.2, *p* = 0.26 for 10-weeks and t-value _(df =20)_ = 1.8, *p* = 0.09. (**C**) Heatmap of scores for each category within the placebo group. The placebo group shows a decline in mental fatigue scores during the open-label phase when compared to baseline scores (t-value _(df =24)_ = 0.43, *p* = 0.67 for 5-weeks; t-value _(df =23)_ = 1.1, *p* = 0.28 for 10-weeks, t-value _(df =23)_ = 2.3, *p* = 0.032 for 15-weeks). For reduced activity: t-value _(df =23)_ = 0.04, *p* = 0.97 for 5-weeks, t-value _(df =22)_ = 0.06, *p* = 0.96 for 10-weeks and t-value _(df =22)_ = 0.8, *p* = 0.43 for 15-weeks. Reduced motivation: t-value _(df =22)_ = 1.67, *p* = 0.11 for 5-weeks, t-value _(df =23)_ = 1.5, *p* = 0.14 for 10-weeks and t-value _(df =23)_ = 1.5, *p* = 0.15 for 15-weeks). *p ≤ 0.05 and ** *p* ≤ 0.01.

There was a significant main effect of the OEA supplementation on the total MFI-20 score (F_(1,180)_ = 6.5, *p* = 0.01 (adjusted *p* = 0.03), Fig. 3A) indicating randomized group differences on fatigue over the 15-week study period. There was no main effect of time and no interaction between intervention and time (*p* > 0.70) for the total MFI-20 score. For the subscale analyses, there was a significant main effect of OEA supplementation on general fatigue (F_(1,183)_ = 3.9, *p* = 0.05), mental fatigue (F_(1,179)_ = 7.8, *p* = 0.006), reduced activity (F_(1,182)_ = 5.7, *p* = 0.018) and reduced motivation (F_(1,182)_ = 6.7, *p* = 0.01). There was no main effect of time, and no interaction between OEA intervention and time for any of these measures (*p* > 0.1). Paired t-tests evaluating within-group changes showed that participants in the OEA group had significantly lower scores (indicating better performance) for total fatigue and for the subcategories of general fatigue and mental fatigue for across all study visits throughout the RCT and the open-label phases compared to the baseline (see Fig. 3B). During the open-label phase, scores for mental fatigue within the placebo group were significantly lower compared to baseline (see Fig. 3C). A heatmap of individual questions on the MFI-20 test showed lowered scoring on various fatigue related questions among Veterans who received OEA compared to those who received a placebo (supplementary Fig. 1).

**Fig. 4:**
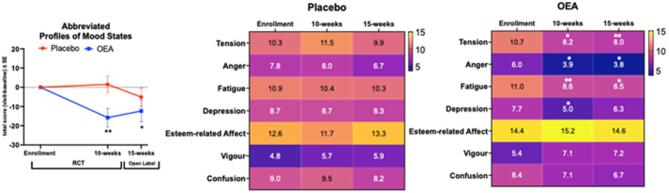
Total mood disturbances and subscale scores on POMS showed improvements in mood and vigor-related outcomes following OEA supplementation. Total Mood Scores were scaled to 100. (**A**) Following MLM analysis for the main effects of OEA intervention (F_(1,137)_ = 9.3, *p* ≤ 0.05), subsequent paired t-test analyses (mean ± SE, placebo *n* = 23; OEA *n* = 21) were performed to ensure we can examine within person change for each subject. Total Mood Scores for each visit are subtracted from baseline, where a lowering of the scores indicates improvement. For the OEA group, change between each visit and baseline was significant for the OEA group (t-value _(df =22)_ = 3.2, *p* = 0.004 for 10-weeks and t-value _(df =20)_ = 2.2, *p* = 0.04 for 15-weeks. There were no differences in change between baseline and each visit for the placebo group (t-value _(df =23)_ = −0.35, *p* = 0.73 for 10-weeks and t-value _(df =23)_ = 1.1, *p* = 0.29 for 15-weeks). (**B**) Heatmap of scores for each subscale. Paired t-test showed lowering of scores for tension, anger, and fatigue were significant for the OEA group at each of the study visits (tension: t-value _(df =22)_ = 2.7, *p* = 0.014 for 10-weeks and t-value_(df =20)_ = 2.8, *p* = 0.01 for 15-weeks; anger: t-value _(df =22)_ = 2.2, *p* = 0.037 for 10-weeks and t-value _(df =20)_ = 2.2, *p* = 0.042 for 15-weeks; fatigue: t-value _(df =22)_ = 3.2, *p* = 0.005 for 10-weeks and t-value _(df =20)_ = 2.3, *p* = 0.031 for 15-weeks and depression: t-value _(df =22)_ = 2.3, *p* = 0.030 for 10-weeks and t-value _(df =20)_ = 1.2, *p* = 0.32 for 15-weeks; esteem related effects: t-value _(df =22)_ = −1.9, *p* = 0.067 for 10-weeks and t-value_(df =20)_ = −0.221, *p* = 0.83 for 15-weeks). (**C**) Heatmap of the for each category within the placebo group. There were no significant differences for any of the paired t-test analyses for the POMS sub scales. *p ≤ 0.05 and ** *p* ≤ 0.01.

There was a main effect of OEA on the TMD scores for abbreviated POMS (F_(1,135)_ = 6.9, *p* = 0.01 (adjusted *p* = 0.03), Fig. 4A). However, there was no main effect of time (*p* = 0.14) and no interaction between time and intervention (*p* = 0.16) for TMD scores. For the abbreviated POMS subscales, there was a significant main effect of OEA for anger (F_(1,137)_ = 9.3, *p* = 0.003), esteem-related affects (F_(1,135)_ = 7.9, *p* = 0.006) and confusion (F_(1,137)_ = 5.3, *p* = 0.023). There was no main effect of time (*p* > 0.05) and no interaction between time and treatment for any of these measures (*p* > 0.05). While there were no main effects of OEA intervention or time (*p* > 0.10), there was a significant interaction between time and OEA intervention for tension (F_(2,89)_ = 5.5, *p* = 0.005). Similarly, there was no effect of OEA intervention and time (*p* > 0.10) but there was an interaction between OEA and time for the depression subscale(F_(2,91)_ = 3.2, *p* = 0.044). For fatigue, there was only a significant effect of time (F_(2,76)_ = 3.8, *p* = 0.026) but no effect of OEA intervention and no interaction between OEA intervention and time (*p* > 0.14). There was no significant effect of OEA, no effect of time and no interaction between time and OEA (*p* > 0.1). Within-group analyses using the paired t-tests showed significantly lower scores (indicating better performance) for tension, anger and fatigue at both the end of the RCT and open-label phases compared to baseline in the OEA group. There were no differences from baseline to any of the visits for the placebo group for any of these measures (see Fig. 4B and C).

### Intervention with OEA improved several domains of the quality of life

**Fig. 5:**
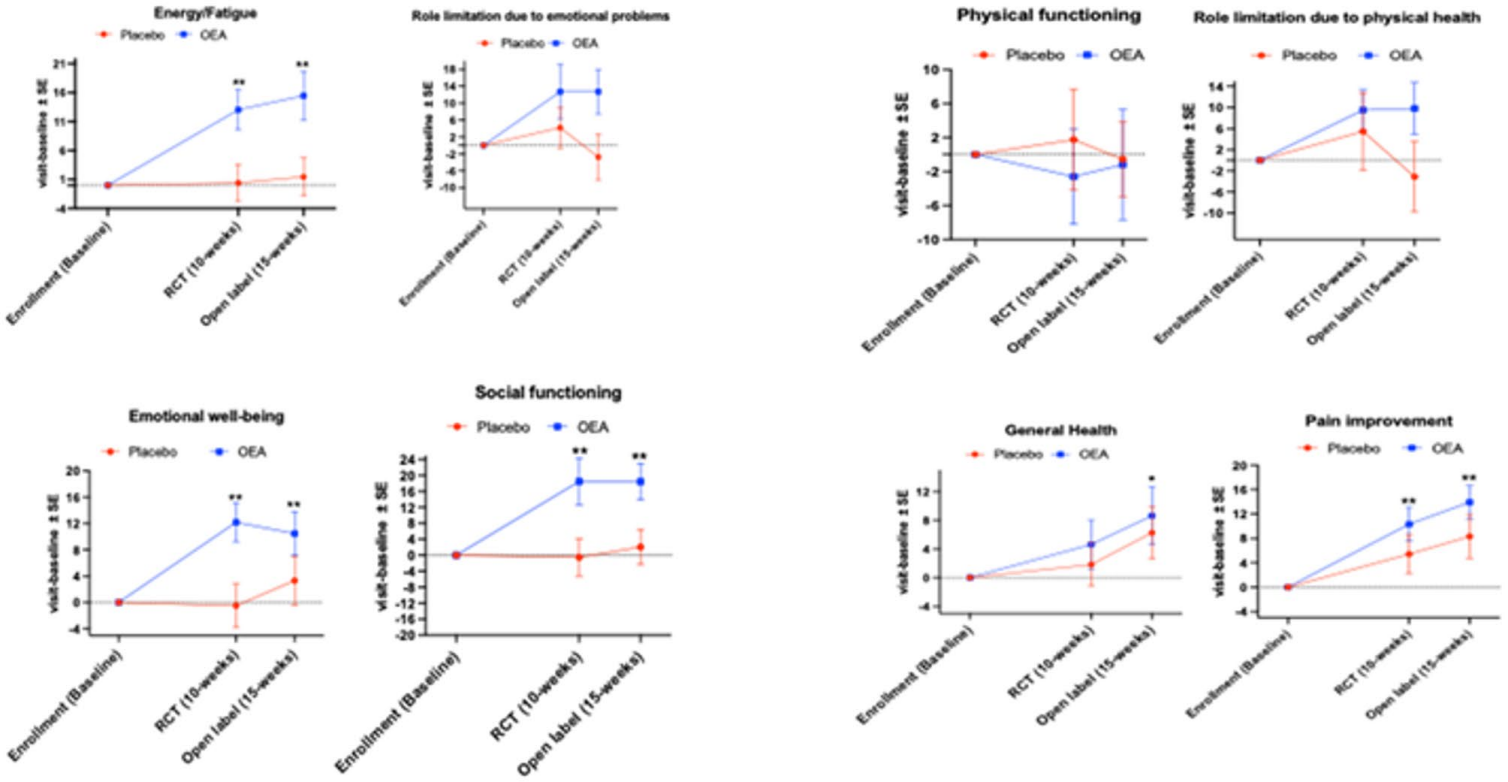
Sub-scales of SF-36 V showed the main effects of OEA intervention for improvements in energy/fatigue, emotional well-being and social functioning scores. (**A**) Mean ± SE, placebo *n* = 23; OEA *n* = 21. An interactive effect of time and OEA was significant for energy/fatigue (F_(2,88)_ = 4.8, *p* ≤ 0.05), emotional well-being (F_(2,87)_ = 3.2, *p* ≤ 0.05), and social functioning (F_(2,88)_ = 3.3, *p* ≤ 0.05). Paired t-test analyses showed that for the OEA group, there was a significant increase in the total score from baseline at both the RCT and the open label phase for energy/fatigue (t-value _(df =22)_ = −3.8, *p* = 0.001 for 10-weeks and t-value _(df =20)_ = −3.7, *p* = 0.001 for 15-weeks), emotional well-being (t-value _(df =22)_ = −4.1, *p* < 0.001 for 10-weeks and t-value _(df =20)_ = −3.2, *p* = 0.005 for 15-weeks), social functioning (t-value _(df =22)_ = −3.2, *p* = 0.004 for 10-weeks and t-value _(df =20)_ = −4.1, *p* < 0.001 for 15-weeks), general health (t-value _(df =22)_ = −1.4, *p* = 0.19 for 10-weeks and t-value _(df =20)_ = −2.1, *p* = 0.045) for 15-weeks) and pain (t-value _(df =22)_ = −3.7, *p* = 0.001 for 10-weeks and t-value _(df =20)_ = −5.1, *p* < 0.001). For the placebo group, there was a significant change from baseline to 15-weeks for pain (t-value _(df =23)_ = −1.7, *p* = 0.1 for 10-weeks and t-value _(df =20)_ = −2.3, *p* = 0.03). There were no other differences observed for the paired t-test analyses. (**B**) Figures show the group differences for role limitation due to physical health, role limitation due to emotional problems, pain and general health. *p ≤ 0.05 and ** *p* ≤ 0.01.

There were no baseline differences between the OEA and the placebo groups for physical functioning, role limitation due to physical or mental health, energy/fatigue, emotional well-being, social functioning, pain or general health on the SF-36 V form (Supplementary Table 1). For energy/fatigue, there was a significant effect of time (F_(2,88)_ = 6.6, *p* = 0.002), no effect of intervention (*p* = 0.36) and an interaction between time and OEA intervention (F_(2,88)_ = 4.8, *p* = 0.011). For emotional well-being, there was an effect of time (F_(2,78)_ = 3.4, *p* = 0.05), no effect of OEA (*p* = 0.06) and an interaction between OEA and time (F_(2,87)_ = 3.2, *p* = 0.045). For social functioning, there was an effect of time (F_(2,88)_ = 3.6, *p* = 0.03), no effect of OEA (*p* = 0.07) and an interaction between time and OEA intervention (F_(2,88)_ = 3.3, *p* = 0.04). For pain, there was no effect of OEA (*p* = 0.31), an effect of time (F_(2,89)_ = 9.9, *p* < 0.001) and no interaction between time and OEA (*p* = 0.5). Similarly, for general health, there was no effect of OEA (*p* = 0.2), an effect of time (F_(2,86)_ = 3.6, *p* = 0.03) and no interaction between OEA and time (*p* = 0.8). Within group analyses using the paired t-tests showed that in the OEA group, compared to baseline, there was a significant increase in scores (indicating better performances) for energy/fatigue, emotional well-being, and social functioning at the end of the RCT and the open-label phases (see Fig. 5A and B). A heatmap of scores for individual questions by intervention assignment is shown in the supplementary Fig. 2.

### No effect of OEA intervention on cognitive performance or pain over the 15-week intervention period

There was no main effect of OEA intervention on cognition as quantified by the sum of index scores for RBANS (Supplementary Fig. 3 A). When examining the subscales, there was a significant intervention effect for language (F_(1,121)_ = 6.8, *p* = 0.01) but no effect of time (*p* = 0.8) and no interaction between time and intervention (*p* = 0.4). For immediate memory, there was a significant effect of the intervention (F_(1,135)_ = 5.8, *p* = 0.02) and an effect of time (F_(2,88)_ = 3.6, *p* = 0.03). However, there was no interaction between time and intervention for this measure (*p* = 0.9, Supplementary Fig. 3 A). There were no significant effects on any other subcategories of the RBANS test (Supplementary Fig. 3B and Supplementary Fig. 3 C). Analysis using the paired t-test showed that, at 10-weeks, both the OEA and placebo groups showed improvements compared to their baseline visits. For language, no significant changes were seen at any of the visits when compared to baseline in either the OEA or the placebo group. For the CNS Vital Signs test, there were no group differences between the two intervention arms for the NCI (Supplementary Fig. 4 A and Supplementary Fig. 4B). There were also no effects of OEA intervention for any of the subscales of the CNS Vital Signs test (Supplementary Fig. 4 A and Supplementary Fig. 4B). There were no effects of OEA intervention on the self-report of pain, as assessed by the total scores on the SF-MPQ (Supplementary Fig. 5).

### Similar adverse event reporting outcomes were observed in the OEA and placebo groups

Table 2 shows the total number of AEs by severity and relatedness to the study intervention reported by 19 individuals in the placebo group and 18 individuals in the OEA group. Table 3 shows the occurrence of the types of AEs in each group over the study duration (see Supplementary Table 2 for the detailed list of AEs). There were no significant differences in the types of AEs between the placebo and OEA groups. There was one withdrawal from the study in the OEA group due to self-reported exacerbation of PTSD. There was also a self-report of exacerbation of PTSD during the RCT in the placebo group in one participant. These events were deemed unlikely to be related to OEA as per the investigating physician. For musculoskeletal problems, there were five events in the placebo group and three events in the OEA group. For gastrointestinal (GI) complaints, there were a total of 11 events in the placebo group and 12 in the OEA group, of which 10 occurred in the placebo group and 8 occurred in the OEA during the RCT (*p* > 0.05). There were no serious adverse events and safety labs did not reveal any clinically significant safety concerns.

**Table 2:** Adverse event presented by severity and relatedness to the study intervention.

Adverse events		
	Placebo (*n* = 26)	OEA (*n* = 26)
Events n (participant n)	51 (19)	53 (18)
Not-related	22 (10)	11 (6)
Mild	18 (9)	10 (5)
Moderate	4 (3)	1 (1)
Severe	0 (0)	0 (0)
Unlikely-related	16 (10)	27 (10)
Mild	12 (9)	12 (5)
Moderate	4 (2)	15 (7)
Severe	0 (0)	0 (0)
Possibly-related	11 (8)	11 (6)
Mild	9 (7)	9 (5)
Moderate	2 (2)	2 (2)
Severe	0 (0)	0 (0)
Probably-related	2 (2)	4 (3)
Mild	2 (2)	4 (3)
Moderate	0 (0)	0 (0)
Severe	0 (0)	0 (0)

**Table 3:** Types of AE experienced by the participants during the trial.

Number of participants (%)	Placebo (*n* = 26)	OEA (*n* = 26)
Abnormal labs	1 (2.6)	2 (5.7)
Cognitive	2 (5.1)	0 (0.0)
Cold/flu/COVID19	4 (10.3)	1 (2.9)
Fatigue	1 (2.3)	2 (5.4)
Gastrointestinal	11 (25.6)	12 (32.4)
Genitourinary	3 (7.0)	0 (0.0)
Musculoskeletal	5 (11.68)	3 (8.1)
Neurological	1 (2.3)	2 (5.4)
Psycho-affective	2 (4.7)	3 (8.1)
Other	12 (27.9)	10 (27.0)
Sleep	1 (2.3)	2 (5.1)
Total	43 (100%)	37 (100)

### Plasma OEA levels were increased with supplementation

**Fig. 6:**
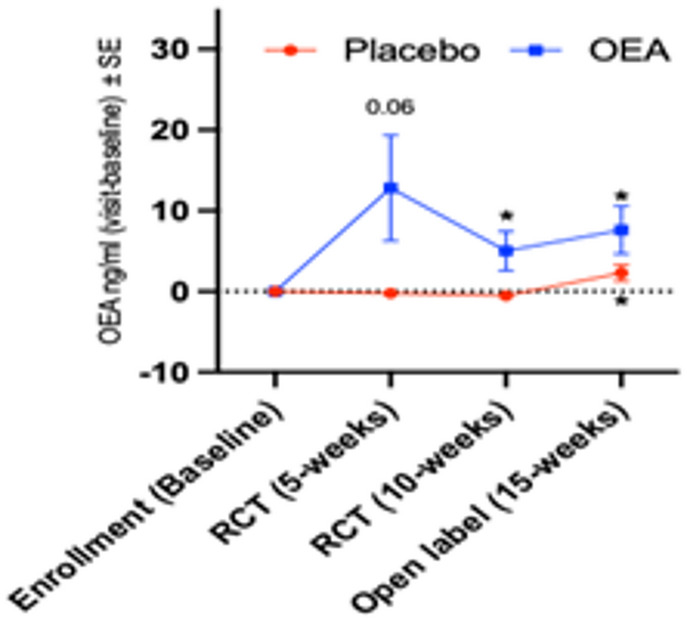
Plasma OEA levels were increased in the intervention group compared to the placebo group. Mean difference at each visit compared to baseline ± SE (placebo *n* = 23; OEA *n* = 21). Compared to baseline, concentrations of OEA increased at each subsequent study visit (t-value _(df =22)_ = −1.97, *p* = 0.061 at 5-weeks, t-value _(df =2)_ = −2.1, *p* = 0.049 at 10-weeks and t-value _(df =18)_ = −2.2, *p* = 0.037). Levels of OEA significantly increased during the 15-week open label phase (t-value _(df = 22)_ = −2.2, *p* = 0.040). *p ≤ 0.05 and ** *p* ≤ 0.01.

There were no significant differences at baseline between the placebo and the OEA groups for any of the ethanolamides measured in this study (see Table 1). Self-reported use of cannabis did not differ between the placebo and the OEA groups (*n* = 3 per group) and self-reported cannabis use was not associated with levels of any of the plasma ethanolamides. There was a significant main effect of OEA intervention on plasma OEA levels (F_(1,78)_ = 10.2, *p* = 0.002), there was an effect of time (F_(3,86)_ = 5.5, *p* = 0.002), and an interaction between time and OEA intervention (F_(3,86)_ = 3.7, *p* = 0.015). However, there was no significant effect of OEA supplementation on plasma AEA levels (Supplementary Figs. 6). There was a significant effect of OEA supplementation on EPEA levels (F_(1,165)_ = 4.3, *p* = 0.039) but no effect of time and no interaction between time and OEA supplementation (*p* > 0.05). *Post-hoc* paired t-test showed significant increases in OEA levels from baseline to 10 weeks and 15 weeks in the OEA group and from baseline to the 15 weeks within the placebo group (Fig. 6). However, there were no significant within group differences for plasma EPEA levels in either the OEA or the placebo group (Supplementary Fig. 6 A and Supplementary Fig. 6B) based on paired t-tests. No significant group differences between the OEA and the placebo groups were observed in plasma cytokines, LDL, and TG levels (Supplementary Figs. 7 and Supplementary Fig. 8).

## Discussion

It has been 35 years since the GW, yet many Veterans from this era continue to experience debilitating fatigue and problems with mood, cognition and pain^2^. To date, there are no approved therapies available to improve the quality of life for GW Veterans with GWI. Oleoylethanolamide has been shown to improve energy homeostasis and lipid metabolism, and to reduce inflammation in animal studies and in clinical trials among obese individuals and patients with nonalcoholic fatty liver disease^36,48–50^. Our mouse model studies showed that oral administration of OEA in GW toxicants-exposed mice resulted in improved performance on neurobehavioral tasks that assess cognition, anxiety and fatigue, which corresponded to reduced inflammation and lipid dysfunction in the brain^31^. In this pilot and small exploratory study in Veterans with GWI, conducted over a 15-week period, OEA was found to be safe, improved mood and reduced fatigue, which corresponded with improved general health and well-being, without impacting cognition or pain.

At baseline, Veterans with GWI had average fatigue scores consistent with scores of individuals experiencing significant fatigue^51,52^. By the conclusion of the RCT and the open-label phases, the OEA group demonstrated a reduction in the total MFI-20 score, equating to a decrease of approximately 8 points. This change aligns with thresholds for clinical significance, underscoring the potential real-world impact of OEA in improving fatigue-related outcomes^53^. Further analysis of the five fatigue domains captured by the MFI-20 test revealed that OEA consistently reduced general, physical, and mental fatigue over the trial duration. This suggests that OEA may exert a broad-spectrum effect, addressing both the physical manifestations of fatigue, such as reduced energy and stamina, as well as mental exhaustion. Mental fatigue also decreased in the placebo group during the open-label phase, suggesting a possible improvement after OEA consumption within 5 weeks.

The Profile of Mood States focuses on temporary mood states rather than stable personality traits. As such, the POMS provides valuable insights into emotional well-being and the psychological impact of various interventions^54,55^. A significant reduction in tension and anger scores suggests that OEA may alleviate stress, nervousness, and irritability. A reduction in fatigue highlights a potential enhancement in energy and alertness, which is a key indicator of a positive mood state. Collectively, these changes suggest that OEA supplementation effectively reduces negative emotional states and promotes positive ones, leading to an overall improvement in psychological health, as reflected by a reduction in the TMD score. The findings from the SF36V suggest that the OEA intervention had a positive impact across several domains of health and well-being, with some differences between the RCT phase and the open-label phases. Among those receiving OEA throughout the trial, there were increases in the energy/fatigue, emotional well-being and social-functioning scores from baseline during both phases, highlighting that these areas were consistently improved by the OEA intervention. Physical activity limitations were significantly reduced during the RCT phase in the OEA group.

In contrast, limitations due to emotional health and general well-being were significantly reduced during the open-label phase. This suggests evolving benefits of the intervention with time or differences in participant perceptions and experiences under the two study conditions. There was a significant improvement in pain perception during both the RCT and the open-label phases; however, pain, as quantified using the SF-McGill pain questionnaire, did not show an improvement. This suggests that factors such as expectations or behavioral adjustments may have played a role in pain perception. These findings indicate that while OEA intervention positively impacts both physical and emotional health, enhancing participants’ ability to engage socially and improving their overall quality of life, there was no self-reported improvement in pain.

Adverse event occurrences were similar between the placebo and OEA groups, supporting the safety profile of OEA in this GW Veteran population. The absence of significant differences in types of AEs across intervention groups suggests that OEA was well-tolerated. This finding is particularly important given the chronic nature of GWI and the potential requirement for long-term supplementation with OEA. The single withdrawal due to an exacerbation of PTSD occurred during the RCT within the OEA group, and this AE was also reported by a participant in the placebo group, also during the RCT phase, which did not result in withdrawal from the study. New trials evaluating OEA in patients with PTSD may be warranted, in light of recent reports showing alterations in the endocannabinoid system in PTSD and potential beneficial effects of its modulation in rodent models of PTSD^56,57^. Overall, these results reinforce the safety of OEA supplementation for Veterans with GWI/CMI over a short 15-week period but still provide valuable guidance for its use as a dietary supplement in the future.

Despite the limitation of non-fasting blood collection, we observed an increase in OEA levels compared to placebo during the RCT and significant increases in the placebo group from baseline during the open-label period. However, there were no significant differences in any of the other ethanolamides. Since prior studies have shown that fasting vs. lipid infusion did not have a significant effect on plasma OEA levels, we anticipate that fasting is unlikely to affect the OEA increases observed in the OEA intervention group^58^. Prior studies have also shown that high-intensity-interval training can increase plasma OEA levels^59^. Elevated OEA concentrations have also been reported in the plasma of individuals with depression and in alcohol binge drinkers^60^, although these increases were modest, reaching only about 1 ng/ml. Because we did not specifically collect data on physical activity or other behavioral factors, we cannot exclude the possibility that such variables may have influenced plasma OEA levels. However, the increases observed in our study, ranging from 5 to 13 ng/ml following OEA supplementation, far exceeded those reported in these prior studies. Moreover, the placebo-controlled design of our trial would have minimized any potential confounding effects by distributing such factors randomly between the two intervention groups. Therefore, it is unlikely that differences in physical activity or behavior features account for the observed increases in plasma OEA concentrations. Future investigations are still needed to determine the influences on plasma OEA levels among Veterans with GWI to better design the OEA supplementation strategies. These data suggest that the observed effects were likely related to OEA itself, rather than through modulation of other ethanolamides, particularly AEA, levels which then target the cannabinoid receptors. Additionally, there were no significant differences between the OEA and the placebo group in blood LDL and TG levels. Therefore, the observed clinical findings are unlikely to be attributable to OEA’s effects on satiety, as no significant weight loss was observed with OEA intervention. Similarly, a lack of change in blood cytokines suggests that alterations in peripheral immune responses may not be involved in modulating the observed effects. However, given that non-fasting samples were used, food may have impacted these observations.

This clinical trial had a limited sample size of 52 GW Veterans with GWI and was performed over a short period. The exploratory nature of the trial necessitated the use of a small sample size, particularly given the fact that recruiting GW Veterans in scientific research continues to be a challenge due to significant barriers including mistrust of government studies, chronic health issues, and logistical challenges such as limited access to research centers and transportation. These factors, combined with concerns about stigma and privacy, limit their participation in research studies. Another limitation of the study was that analysis of several subscales for MFI-20, POMS, CNS-Vital Signs test, RBANS and SF-36 V tests were performed without multiple testing corrections. Nonetheless, despite the small sample size and the multiple testing correction limitations, the data collected offer valuable exploratory insights to inform future studies and recruitment strategies. Designing subsequent trial with larger sample sizes is required to more fully test and confirm the results from this small pilot study. Designing larger trials will require significant investment in engaging GW Veteran advocates and using GW Veteran networking to help with encouraging GW Veterans to participate in these studies. The study’s open-label phase, during the last 5-weeks, may have introduced potential biases related to participant expectations. However, since the effects of OEA were detected during the blinded RCT period, placebo effects were unlikely to be a factor within the first 10-weeks of the trial. As such, the randomized placebo-controlled trial design helped mitigate bias by providing a robust framework for evaluating OEA supplementation under controlled conditions.

It is also possible that the lack of observed changes in pain and cognitive performance may be inherent to the multifaceted nature of GWI, pointing to the likelihood that separate biological processes contribute to different symptom clusters. While our preclinical mouse model studies suggested improvement in learning and memory, this was not replicated in the current clinical study. In the mouse study, OEA was administered for 6 months, whereas the current study examined OEA intervention for only 15 weeks. The brief treatment period, accompanied by practice effects with neurological testing^61^, may have constrained the detection of changes in cognitive outcomes, which might necessitate longer interventions or more sensitive assessment tools. The RBANS test is a screening measure and may not possess the adequate sensitivity to detect subtle cognitive decrements in memory consolidation and retrieval and the range to detect subtle improvements post-intervention. Cognitive characterization of the Boston Biorepository, Recruitment and Integrated Network (BBRAIN) cohort showed that Veterans with GWI demonstrated significantly poorer performance than controls on verbal learning tasks on the California Verbal Learning Test-II (CVLT-II), with deficits linked to neurotoxicant exposures such as pesticides and nerve agents^62^. As such, CVLT-II can provide indices for encoding vs. consolidation deficits as well as help assess memory retrieval. Therefore, CVLT-II may be more suitable than the RBANS for assessing cognition in GWI and is also recommended in the GWI common data elements^63^.

The factors governing cognitive dysfunction in GWI remain to be fully elucidated and may require a more mechanistic approach to understand cognitive dysfunction and disruptions in neuronal networks in various brain regions that are affected in Veterans with GWI^64–66^. The use of a single fixed dose limits the understanding of dose-response relationships. Therefore, future trials with multiple ascending doses or multiple doses as separate arms of the trial would help test the dose-response relationship. The absence of biomarkers of pharmacodynamics and efficacy of OEA limits further interpretations of this study, and additional work is required to identify such biomarkers to support further translational studies of OEA in GWI.

Several plausible biological mechanisms may govern the effects of OEA on improving mood and reducing fatigue. These should be explored in future studies to further evaluate aspects of target engagement. For instance, it is well known that OEA regulates energy balance, inflammation, and neural signaling, primarily through PPARα activation^67^. It is therefore possible that by promoting mitochondrial bioenergetics function, OEA enhances cellular energy availability and reduces metabolic inefficiencies, thereby reducing fatigue and improving overall energy levels. Its anti-inflammatory properties help improve general activities of daily living by promoting positive mood and energy states. Future studies should correlate biomarkers with clinical measures to determine which aspects of OEA administration reflect its ability to activate PPARα. Oleoylethanolamide may support stress resilience mediated through neurochemical pathways involving serotonergic and/or dopaminergic systems which are secondary effects of PPARα activation^68–70^. Alternatively, these effects of OEA may be via activation of vagal afferent signaling at the gut-brain interface^17^. Furthermore, OEA contributes to gut health and appetite regulation, while potentially optimizing sleep-wake cycles^5,71,72^, all of which could have a positive impact on general health and activities of daily living for individuals experiencing CMI. As such, further mechanistic studies are required to better understand the role of OEA in maintaining healthy mood and energy states among individuals diagnosed with CMI.

## Conclusion

In this exploratory pilot study, OEA demonstrated a favorable safety profile and showed an ability to reduce fatigue and improve mood and energy levels, highlighting the impact of OEA on both physical and psychological health. Further studies are warranted to elucidate the precise mechanisms underlying OEA’s effects and to confirm its efficacy in larger and diverse GW Veterans populations with GWI and CMI. Given that preclinical studies have shown that OEA targets bioenergetics, neuroinflammation and associated pathways, future clinical studies should aim to confirm these effects of OEA supplementation. Additionally, investigating potential biomarkers of response could help identifying individuals most likely to benefit from OEA, further optimizing its application in managing fatigue and enhancing quality of life in Veterans with GWI. The favorable safety profile of OEA, combined with its ability to alleviate physical and mental fatigue and boost mood and energy levels, underscores the positive impact of OEA on both physical and psychological health.

## Supplementary Information

Below is the link to the electronic supplementary material.

Supplementary Material 1

## Data Availability

The de-identified datasets used and/or analyzed during the current study available from the corresponding author on a reasonable request.

## Declarations

### Disclaimer

The opinions or assertions contained herein are the private views of the authors and do not reflect the views of the Department of Veterans’ Affairs or the Department of Defense.
