# Supplementary material for: Oleoylethanolamide supplementation improves mood and reduces fatigue in veterans with GWI in a 15-week randomized, double-blind, placebo-controlled exploratory clinical trial
Source: Sci Rep. 2026 Jan 9;16:4933. doi: 10.1038/s41598-026-35168-3 (PMC12873323; doi:10.1038/s41598-026-35168-3)
Supplement: Supplementary file 1 — Supplementary Material 1 [file 41598_2026_35168_MOESM1_ESM.pdf]

Supplementary methods:

Post-hoc power calculations. *post-hoc* power was calculated for total scores of the CNS vital signs test, RBANS, MFI-20 and POMS. The differences in means and associated standard deviations (SD) between the baseline and the end of the RCT observed at the current sample size showed that the effect size (Hedges' g) was 0.53 for the MFI-20 total scores, which provided a power of 96% at α = 0.05. For the TMD score on POMS, Hedges' g was calculated to be 0.65, providing a power of 99% at α = 0.05. For RBANS, the sum of index scores, Hedges' g was calculated to be 0.33, which provided a power of 65% at α = 0.05. For the CNS vital signs test, the neurocognitive index (NCI), Hedges' g was 0.07, providing extremely low power of 7% at α = 0.05. For SF36V effect size calculations were limited to energy/fatigue domain; Hedges' g was 0.76, providing power of >99% at α = 0.05.

Supplementary Table 1: Baseline characteristics of subscales of SF36V

	Placebo (n= 26)	OEA (n = 26)
	Mean (SE)	
physical functioning	46.7 (4.8)	55.8 (4.2)
Role limitations due to physical health	40.6 (5.1)	40.1 (5.2)
Role limitations due to emotional problems	47.8 (5.6)	44.9 (5.1)
Energy/Fatigue	28.9 (4.3)	24.8 (4.1)
Emotional well-being	48.3 (4.3)	51.4 (4.1)
Social functioning	45.7 (6.4)	47.6 (5.8)
Pain	35.6 (4.0)	38.1 (4.0)
General Health	34.1 (4.7)	40.9 (4.7)

Supplementary table 2: Types of AE reported

Types	Counts
**Gastrointestinal**	**41**
Abdominal cramping	1
Bloating	1
Constipation	3
Decreased hunger	1
Diagnosed with Celiac Disease	1
Diagnosed with Diverticulosis	1
Diagnosed with GERD	1
Diarrhea	7
Exacerbation of constipation	1
Exacerbation of GERD	1
Gassy	1
Loose stool	8
Loss of appetite	3
Nausea	4
Stomach upset	1
Vomiting	2
Decreased bowel movements	1
Increased/frequent bowel movements	3
**Other**	**26**
Accidental IP Overdose	1
Adenopathy	1
Anaphylaxis	1

Edema in legs	1
Exacerbation of melanoma	1
Fever	1
Infection	4
Pre-diabetes	1
Surgery	2
Blood pressure	3
Dental	3
Skin inflammation	4
Injury	3
**Musculoskeletal**	**8**
Back pain	2
Costochondritis	1
Hip pain	1
Joint stiffness	1
Leg cramps	1
Joint pain	2
**Cold/flu**	**6**
COVID-19	3
Flu-like symptoms	1
Head cold	2
**Psychoaffective**	**5**
Exacerbation of PTSD	3
Mood	2
**Abnormal lab**	**4**
Anemia	1

Elevated bilirubin	1
Elevated triglycerides	1
Low absolute lymphocytes	1
**Sleep**	**3**
Insomnia	1
Vivid dreams	2
**Fatigue**	**3**
Fatigue	2
Lethargic	1
**Neurological**	**3**
Headaches	2
Migraine	1
**Genitourinary**	**3**
Incontinence	2
Urinary hesitancy	1
**Cognitive**	**2**
Loss of focus	1
Mental Fog	1

Supplementary Figure 1

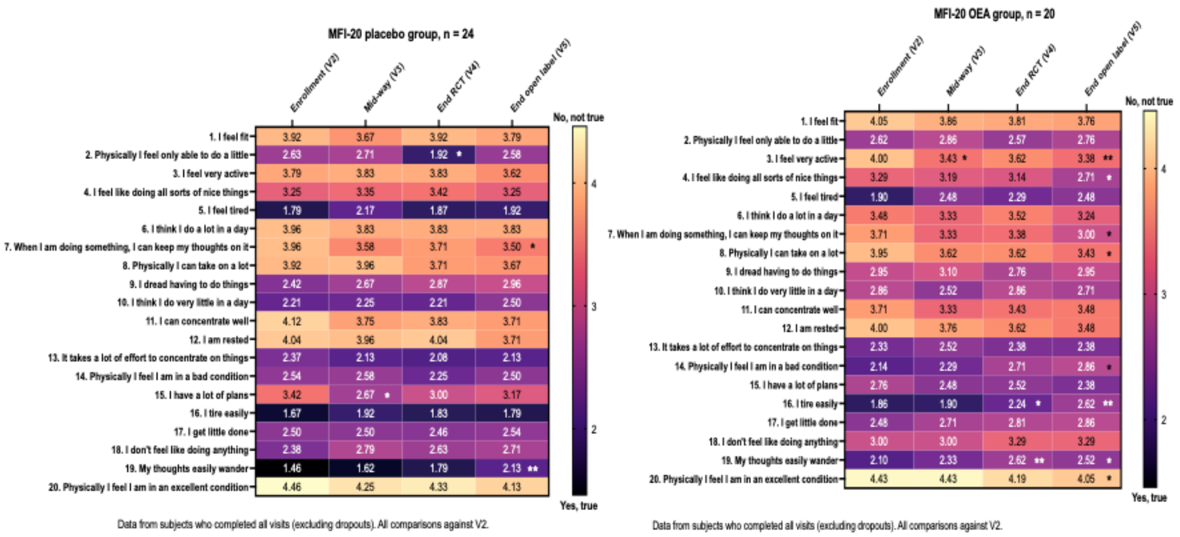

Supplementary Figure 1: A heatmap of individual questions of MFI20 assessment showed improvement in the OEA group over time. The Heatmap of scoring change with time over the RCT and the open label phases for the OEA groups showed continuous improvement in responses over the course of OEA supplementation across fatigue items. Paired-test were performed only on exploratory basis. Statistical significance was most notable from 10 weeks to 15 weeks of OEA supplementation. The heatmap showed no pattern of improvement during the RCT for fatigue items in the placebo group. During the open-label phase, a significant improvement of focus and attention was observed after OEA supplementation at V5 (see questions 7 and 19). *p ≤ 0.05 and ** p ≤ 0.01.

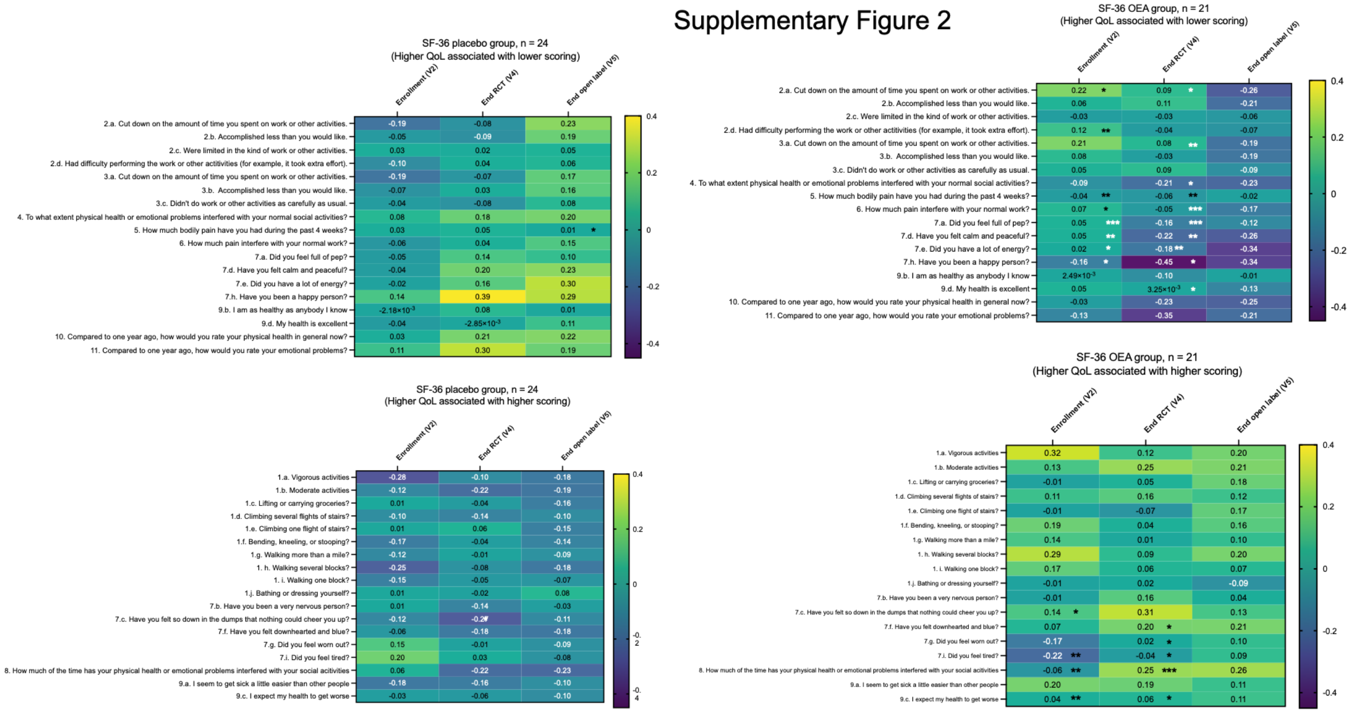

Supplementary Figure 2: A heatmap of individual questions of SF36V quality of life assessment showed improvement in the OEA group over time. There were significant improvements from baseline to each subsequent visit for the OEA intervention group. These questions pertained to self-report of fatigue, energy balance, social functioning and emotional well-being, all showing change in scores consistent with improvement. *p ≤ 0.05 and ** p ≤ 0.01.

Supplementary Figure 3

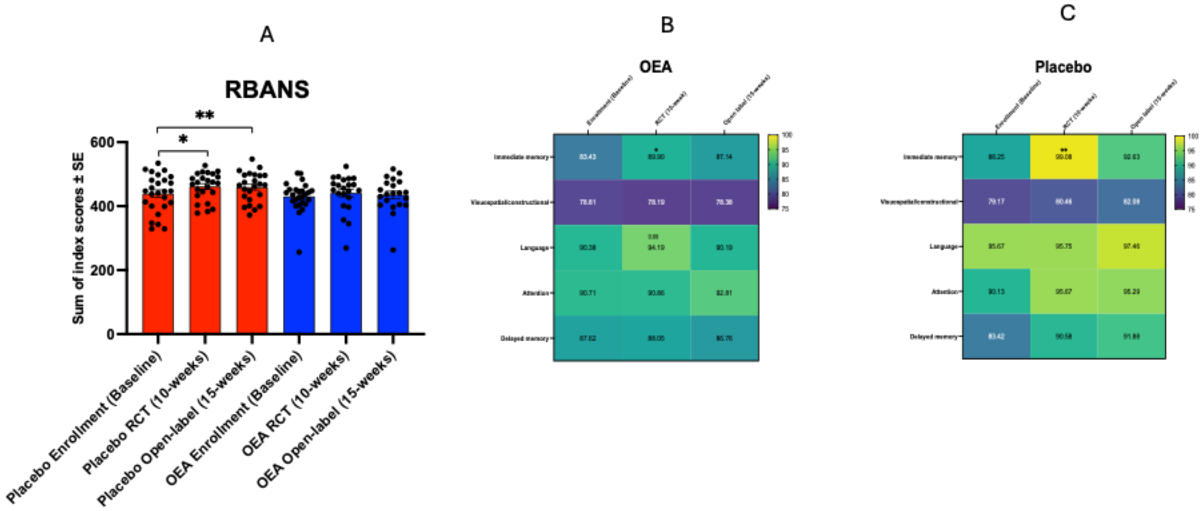

Supplementary Figure 3: There was no improvement in cognition after OEA supplementation as assessed by RBANS. Mean ± SE, placebo (n = 24); OEA (n = 22). (A) There were no significant differences over time in the OEA group for change in the total sum of index for the RBANS test. The placebo effects on cognition were evident within the placebo group which continued to show better scores during the RCT and the open label phase. (B-C) Heatmap of scores of subcategories of cognitive domains evaluated. For immediate memory, significant differences were observed for immediate recall and both placebo and OEA intervention showed better performance at 10-weeks compared to their baseline scores. *p ≤ 0.05 and ** p ≤ 0.01.

Supplementary Figure 4

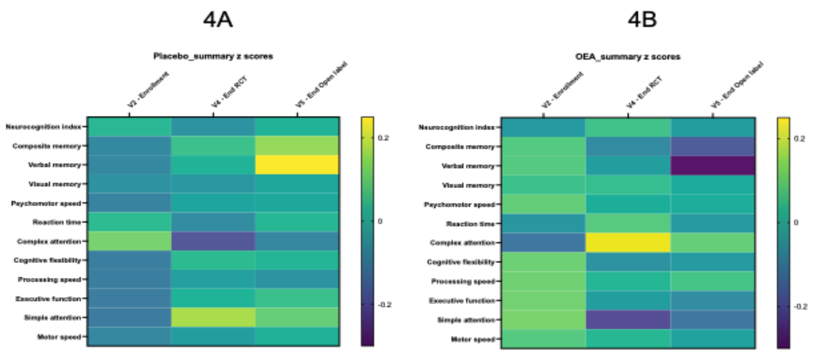

Supplementary Figure 4: A heatmap showing no significant improvement after OEA supplementation for the CNS vital sign test. Mean ± SE, placebo (n = 24); OEA (n = 20). (A) No significant improvements were observed for several parameters of cognition in the OEA group compared to the placebo group. (B) Significant improvement in several cognitive domains were observed for the placebo group over the course of both the RCT and the open-label phase. *p ≤ 0.05 and ** p ≤ 0.01.

Supplementary Figure 5

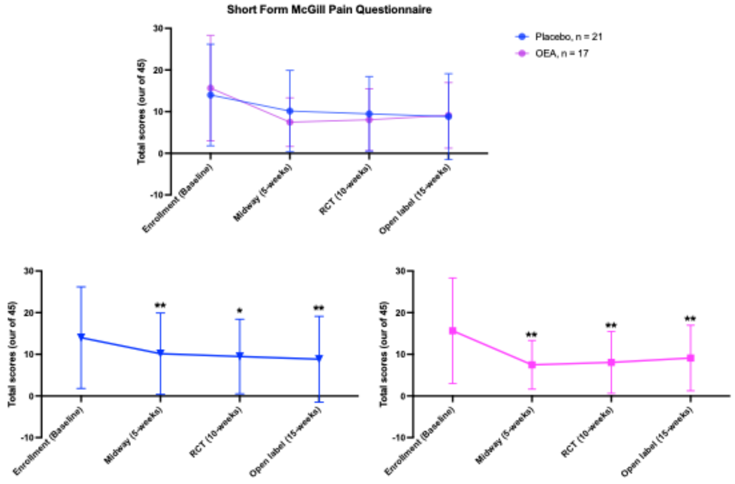

Only includes data from individuals who completed all visits and completed the questionnaire.

Supplementary Figure 5: There were no significant differences between the intervention group for change in pain scores for Short-form McGill pain questionnaire. Mean ± SE, placebo (n = 21); OEA (n = 17). No significant differences were observed between the OEA and the placebo groups for pain scores. In the placebo and OEA groups, a significant decline in the scores was noted at each follow-up visit compared to baseline. *$p \leq 0.05$ and ** $p \leq 0.01$.

Supplementary Figure 6

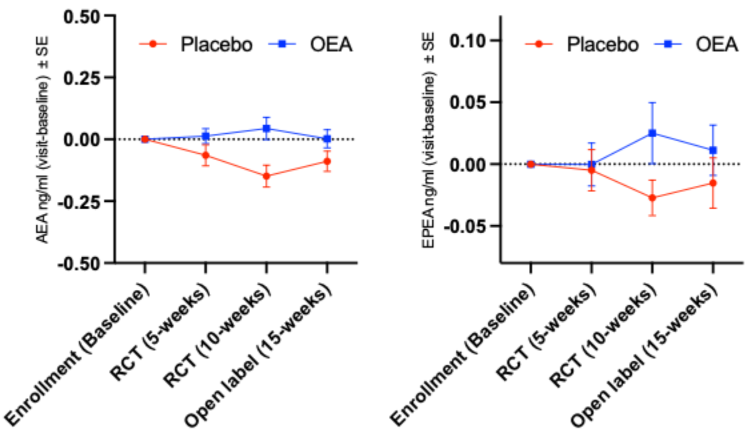

Supplementary figure 6: There was no effect of OEA intervention on AEA or the EPEA levels. Mean ± SE, placebo (n = 22); OEA (n = 18). There were no significant effects of OEA on AEA levels. Concentrations are lower in the placebo control at each visit compared to baseline. While overall MLM showed significant intervention effects of OEA, paired t-test did not show any significant differences between baseline and subsequent visits for the OEA or the placebo group.

Supplementary Figure 7

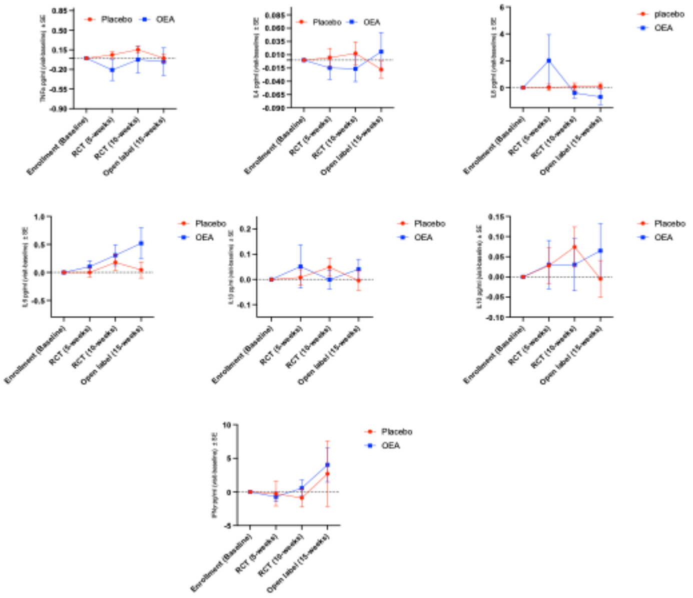

Supplementary figure 7: There was no effect of OEA intervention on blood cytokines. Mean ± SE, placebo (n = 23); OEA (n = 21). There were no significant differences between the OEA and the placebo groups for any of the cytokines measured.

Supplementary Figure 8

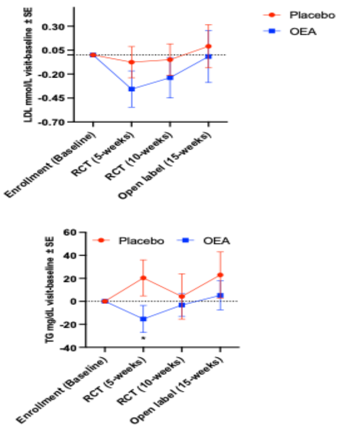

Supplementary figure 8: There was no effect of the OEA intervention on blood LDL and TG levels. Mean ± SE, placebo (n = 24); OEA (n = 21). There were no significant differences between the OEA and the placebo groups for any of the cytokines measured.
